# Study of a COVID-19 induced transition from Face-to-Face to Online Team-Based Learning in Undergraduate Family Medicine

**DOI:** 10.15694/mep.2020.000232.1

**Published:** 2020-10-16

**Authors:** Lisa Jackson, Farah Otaki, Leigh Powell, Ernie Ghiglione, Nabil Zary

**Affiliations:** 1 Mohammed Bin Rashid University of Medicine and Health Sciences (MBRU); 2 LAMS International

**Keywords:** Team-Based Learning, TBL, Family Medicine, Distance Learning, Community of Inquiry, Remote Emergency Teaching

## Abstract

This article was migrated. The article was marked as recommended.

Introduction:

The COVID-19 pandemic has been presenting new challenges for medical schools worldwide. Medical educators are coming-up with creative solutions to address those unprecedented challenges. The purpose of this study is to reflect upon an experience of an online TBL in a Family Medicine Clerkship.

Methods:

This study relied on a qualitative descriptive design. A phenomenological approach was adapted to capture the perception of students regarding online TBL as part of Family Medicine clerkship, using a survey of open-ended questions. The data were inductively analysed using thematic analysis.

Results:

The results of the study revealed that the students, overall, reacted positively to the experience. Five themes of text fragments emerged from the analysis: Equivalency of experience, Efficiency and Ease-of-use, Organization, Novelty, and Design. The students also noted some minor difficulties, that they faced at the beginning of their experience, indicating the existence of a learning curve. They also identified a few opportunities for improvement.

Discussion:

This study demonstrated that combining videoconferencing and lesson delivery software together for TBL enables remote facilitation of the Family Medicine curriculum, and that the students value the experience as such. From the COI perspective, all three factors, namely: social presence, teaching presence, and cognitive presence, are met by such a set-up for distance learning. The students also felt validated and that their voice is heard. The central coordination of the TBL process proved to be crucial to ensure continuity, and also to support individuals’ mental health and team spirit.

Conclusion:

This study concludes that TBL enables rapid transition to distance learning; it promotes analytical and self-directed learning even in extreme circumstances. Moreover, the TBL sessions allow for the facilitators, including the Discipline Lead, to get to know the students, on a personal level, and to monitor and evaluate their performance, over time.

## Introduction

Team Based Learning (TBL) as an educational strategy is used around the world with a research base going back over 30 years (Parmalee *et al*, 2012; Reimschisel *et al.*, 2017). TBL is reported as having advantages over traditional lectures in terms of knowledge acquisition ( Haidet, Kubitz and McCormack, 2014). In medical schools, TBL appears to be reliable, and sustainable ( Thompson *et al,* 2007). It has also been evaluated in medical student clerkship settings in relation to the acquisition of clinical competence, with positive feedback from students ( Figuero-Filho *et al.*, 2015).

TBL draws on the constructivist learning model in which a learner’s existing knowledge is drawn upon to construct new knowledge (Duffy and Cunningham, 1984). Learning is designed to be active, having learners explore relevant problems through small group discussions intended to draw out inconsistencies in learners’ existing knowledge, with the teacher facilitating rather than leading the sessions ( Hrynchak and Batty, 2012). TBL has been evaluated for effectiveness in several pre-clinical and clinical medical training settings. In a pilot study in a neurology clerkship, the performance of students in clinical reasoning exams was found to be better in those given supplementary TBL as compared to those who received traditional didactic input only ( Meike *et al*., 2017). Another study showed that team emotional intelligence improved after using TBL in an internal medicine clerkship, ( Borges *et al*., 2012). A report from the University of Michigan using TBL in a paediatric clerkship demonstrated significant increases in the level of engagement of students being taught with the TBL method as compared with lectures, and scores in examinations also improved when compared with student means on Paediatric Shelf examinations ( Warrier *et al*., 2013).

TBL is widely recognised as a classroom endeavour using a standard approach that relies specifically on certain elements relating to individual preparation and accountability (iRAT), team working and agreement with simultaneous reporting (tRAT and application exercises). Although there are some published experiences using it online, this is mainly related to circumstances such as responding to the need for online courses which are delivered ‘asynchronously’ (i.e., when the students are not all working on the stages of the TBL process at the same time) ( Palsole and Awalt, 2008). In a few other cases, TBL was used online for the purpose of extending the classroom experience using computer aided methods ( Gomez, Wu and Passerini, 2010), or for quality assurance requirement to study a change from off line to online teaching ( Goh, Di Gangi and Gunnells, 2020).

Relevantly, the Community of Inquiry (COI) model has been used extensively to guide the creation of meaningful online and blended learning experiences ( Akyol and Garrison, 2011; Cabrera, Villalon and Chavez., 2017; Garrison and Vaughan, 2007). COI maintains the consideration of three factors: social presence, teaching presence, and cognitive presence, which help to ensure that learning experiences remain valuable, and learners remain connected when shifting the learning environment to other modalities like blended and online learning.

The COVID-19 pandemic presented huge new challenges for medical schools worldwide ( Rose, 2020), where they were given no option but to abruptly transition from classroom setting to online environments). The flip side of the same coin is that the pandemic somehow created opportunities for those schools to test the transferability of their teaching tools and the responsiveness of their curriculums. It also gave medical educators the space to experiment, think out of the box, and come-up with creative solutions to address those unprecedented challenges. From that standpoint, and the Community of Inquiry (COI) model perspective, the purpose of this study is to reflect upon an experience of an online TBL in a Family Medicine Clerkship. To do so, we review the changes adapted to the respective clerkship due to the rapid transition and explore the perceptions of the students of these changes.

## Methods

### Context of the study

**Figure 1.**
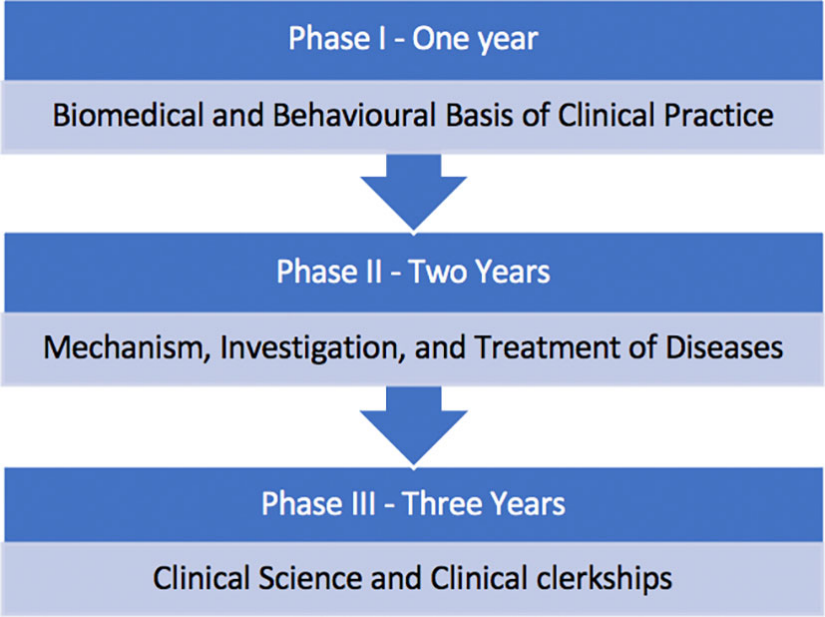
Overview of the Curriculum

The Mohammed Bin Rashid University of Medicine and Health Sciences (MBRU) is in Dubai, United Arab Emirates (UAE). Figure 1 provides an overview of the curriculum of the undergraduate Bachelor of Medicine, Bachelor of Surgery (MBBS) programme at the MBRU College of Medicine (CoM). The first three pre-clinical years (Phases I and II) provide a Basic Science foundation and rely largely on didactic lectures, with some interaction via tutorials, and occasional case-based or team-based teachings. The clinical phase (Phase III) is composed of clinical rotations which span 3 years.

**Figure 2.**
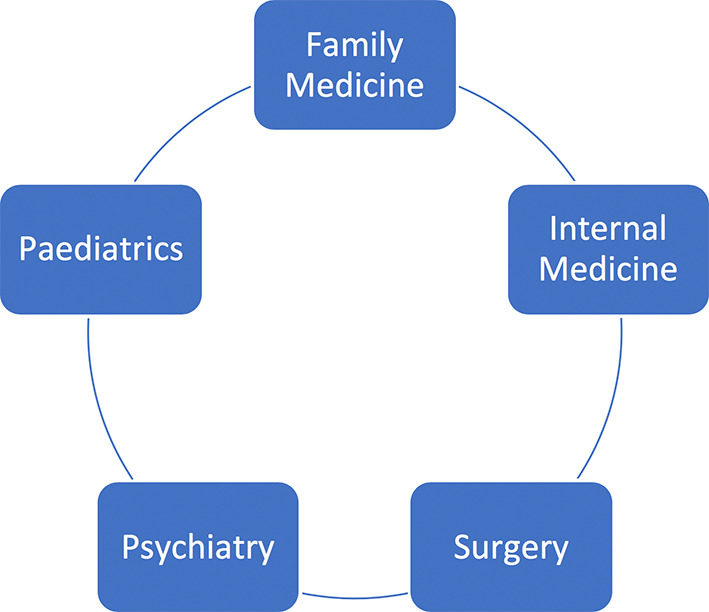
The disciplines represented during year 4 of the MBBS programme

In the fourth year of the programme, Family Medicine is embedded in equal proportion to Internal Medicine, Surgery, Paediatrics, and Psychiatry ( Figure 2). Students spend 4 days a week in clinical settings and rotate through the five disciplines for 8 weeks each over 40 weeks.

## Family Medicine Clerkship under Investigation

### General Description

Family Medicine is provided through a large private hospitals and peripheral clinics group, spread around Dubai, UAE. Starting in September 2019, the CoM implemented the clinical clerkships for the first time.

The core knowledge teaching, corresponding to each clerkship, was provided once per week, over a 4-hour time slot. In the case of Family Medicine, the available time slot lent itself to the concept of TBL, by supporting the concept of teamwork, and the analysis of complex cases, in groups. In addition, the time slot allowed for the joining of multiple Family Medicine Adjunct Faculty who learned the TBL methodology, and in turn contributed to the facilitation of the small group teaching, and delivered specific, predetermined learning material.

### Before the Onset of COVID-19

Prior to starting TBL, each rotation of students received an introductory presentation on what the teaching methodology involved, and the reason for choosing it for Family Medicine teaching. Great care was taken with group formation to ensure balance of local and international students, and of varying expected maturity (from school leavers, all the way to those with work, or other higher education experience). The discipline lead (LJ) ensured there was an Ice Breaker and the implementation of Group Rules for general accountability from individuals and the group.

LJ and one Adjunct Faculty facilitated each TBL session in a large teaching room where there was enough space for 9-10 students to work either together or divided into two smaller groups. Individual Readiness Assessment Test (iRAT) was delivered as a paper exercise at the start of the session, taking usually 10-15 minutes to do 10 Multiple Choice Questions (MCQs) based on prior set reading. Team Readiness Assessment Test (tRAT) then took place in two smaller groups, sitting around the table, and physical scratch cards were used for recording the answers. Papers and scratch cards were collected by LJ for marking and recording of grades for assessment purposes. The MCQs were then discussed, in detail, once both groups had finished the tRAT, in order to iron out any challenges, and/ or address any questions from individuals or groups. This involved going through the incorrect answers to ensure students understood not only the correct answers, but why other answers were incorrect. Students shared their own answers with their teams in the discussion, if they wished. They particularly loved the scratch cards. There were often heated discussions before team decisions were finalized, and excited reactions to right or wrong answers.

The two teams then worked on the application exercise in a single room with one or two facilitators providing guidance in the discussion process. The room was usually noisy. Application exercise results were presented using flip charts or role plays and entailed further questions and discussion. The students often presented mind maps or charts with a sequence for managing the problems, and this could be competitive and lively.

Following the presentation of the teams’ solutions to the application exercises, the Adjunct Faculty and Discipline Lead presented one or two 20 to 30-minute seminars on topics of relevance to the TBL learning outcomes from that day. For example, a session which included an application case involving discussion of prevention and screening, was followed by a summary presentation on immunization through the lifecycle, and the role of primary care in preventing chronic disease. Overall, the TBL sessions took from 3.5 to 4 hours, and were generally focused and intense, with one or two very short breaks.

Between September 2019 and March 2020, three rotations of students had successfully completed all TBL sessions in the live classroom. A fourth rotation had completed two of the required eight sessions in the live classroom when the COVID-19 pandemic supervened and forced all work online.

### After the Onset of COVID-19

A full lockdown in Dubai was implemented on 4 ^th^ April 2020, necessitating the full transition to distance teaching. The fourth rotation group received two weeks classroom TBL, followed by 5 weeks online and the fifth rotation experienced the full teaching programme via online TBL. The onset of the pandemic necessitated the complete exclusion of all faculty, staff, and students from the campus. No lectures or small group teaching on-campus were allowed. Students were also excluded from clinical clerkships in the hospitals and peripheral clinics.

Online teaching was implemented using Microsoft Teams, as video conferencing platform, and combined with an online e-learning platform (Learning Activity Management System, LAMS). Microsoft Teams was used as a virtual classroom while the LAMS platform delivered the learning activities as per the TBL lesson plan.

### Pilot of Online TBL

The first online session of TBL was piloted in mid to late March 2020. At this time, faculty and some staff were still allowed on campus, so faculty came to campus to deliver the session while students attended from home. This allowed piloting of the use of Microsoft Teams with TBL and remote facilitation with the use of software designed for TBL lesson delivery. Several Faculty were in attendance for the first remote session with students at home and Faculty on campus in the teaching room either facilitating and delivering learning material or observing how the process was working.

Prior to the session, EG setup the LAMS system to include the activities, known as sequences in LAMS, to be conducted in the TBL session (i.e., iRAT, tRAT, etc) and added all students to the system, assigned to their individual groups. At the beginning of the session, EJ facilitated an introduction to LAMS, showed the students how to join each sequence and remotely opened the gates as the students progressed through the stages. Initially, this freed up time for LJ to focus on the teaching process and group dynamics. Later, he introduced the discipline lead LJ to the process of moving students through the sequence.

In order to permit the whole TBL process, it was necessary to pilot the use of channels in Teams to ensure students could easily move from a General meeting for the iRAT, to individual channels for the tRAT and application exercises, and then back to General channel for the plenary and discussion. Small group topic teaching relevant to a particular TBL session that day was delivered in the General channel.

### Incorporation of TBL sequence into lesson delivery software

The already developed TBL teaching plans were incorporated into the LAMS sequence by EG without modification by LJ. MCQs for both iRAT and tRAT were transferred to the TBL lesson plan in LAMS and the case study for the application exercises inserted into the sequence after tRAT.

The students completed the MCQ individually online (iRAT) in the General Team channel with EG, LP and LJ in the channels to ensure all went smoothly from the technical standpoint. Electronic scratch card within the software was very similar to the paper version and gave a similar experience as in the classroom.

The students did not experience any major technical difficulties, with most having good internet connections. EG who organized orientation to the LAMS for Faculty and students, was based in Singapore four hours ahead of Dubai, and so the timing of the session in the fourth rotation from April to May was exactly the same as previous rotations, starting at 8:00am (Dubai Local Time). For the fifth rotation, which coincided with the month of Ramadan, one student was based in Sudan two hours earlier than Dubai, and so timing was adjusted to 9:00am (Dubai Local Time) to allow for both fasting and time zone differences.

### Research Design

The investigation revolved around a qualitative descriptive approach to research. Phenomenology was used as the research methodology to understand the lived subjective experience of the participants. As part of this approach, researchers aim to explore how the phenomenon under study is perceived by those who experience and are involved in it first-hand. Ethical approval for the respective teaching initiative was granted by the MBRU, Institutional Review Board (Reference # MBRU-IRB-2019-015).

### Data Collection

All the students enrolled in the fourth and fifth rotations (representing 40% of the total of 47 fourth year students) were invited to participate in a survey, towards the end of their respective rotations. The survey aimed at exploring the participating students’ perception regarding the online TBL, and the transition to distance learning as part of the Family Medicine clerkship. Data were collected via online Microsoft Forms. The survey was composed of the following open-ended questions:

• What were the highlights of the online TBL of the Family Medicine clerkship? (please specify two aspects of your experience)
• What suggestions do you have for improving the application of the online TBL of the Family Medicine clerkship? (please specify two opportunities for improvement)

In order to avoid any biases and to enable the students to express themselves freely, the survey was assembled by a stakeholder who was not involved in the learning and teaching aspects of the experience (FO). Participation in this data collection initiative was completely voluntary. The privacy and the data confidentiality of the students were protected, and no personal identifiers were recorded.

### Study Participants

**Figure 3.**
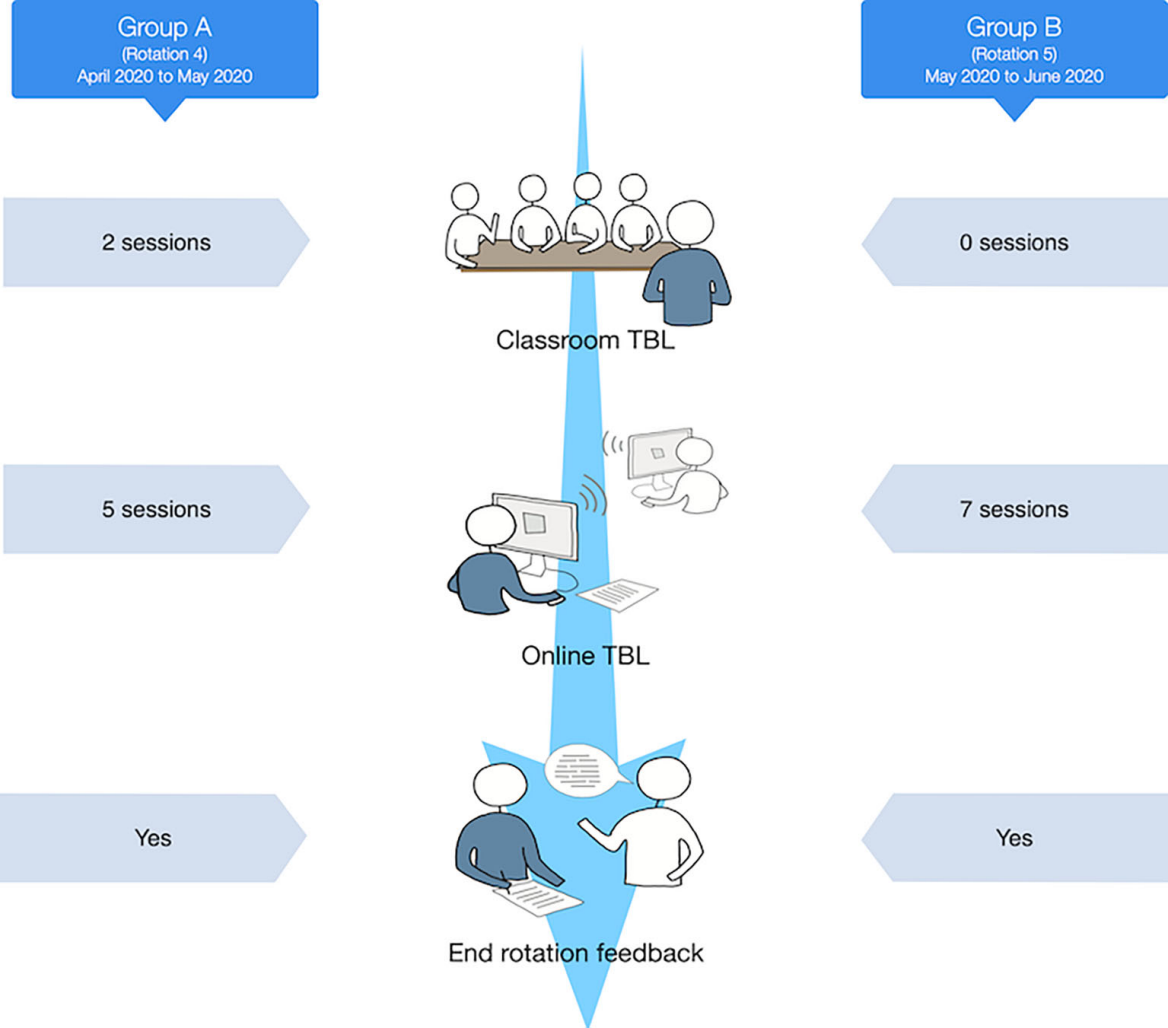
Study Timeline

Out of the 19 students who were invited to participate in this study, 18 students filled the survey (response rate= 95%). The fourth rotation (April to May) was composed of 9 students, who experienced two face-to-face sessions, and five online ones (Group A). As for the fifth rotation (May to June), it was composed of 10 students, who participated in seven online sessions (Group B). The flow of the academic year showing the timing of the study is illustrated in Figure 3.

### Data Analysis

The data analysis took place after the completion of the data collection phase. To structure the data analysis, each participant was assigned a unique identification number (i.e., 1 through 18). The data were thematically analysed using Interpretative Phenomenological Analysis (IPA) ( Braun *et al.*, 2018), through an iterative and inductive process by two researchers (LJ and FO). This interpretative approach enabled capturing the essence of the topic under investigation by describing all its differing aspects. Text fragments that related to the research questions were coded into nodes until no novel insights surfaced, and hence data saturation was attained.

The distinct categories underwent several rounds of reflections, where the different ways by which they might relate to one another were identified. These categories were examined to find the best way to merge them into higher order themes.

## Results/Analysis

**Figure 4.**
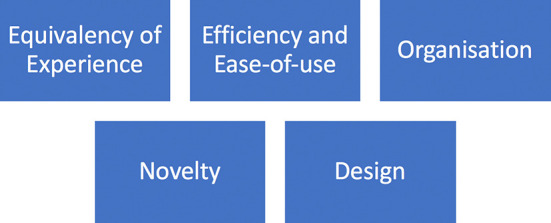
The study’s conceptual framework

The results of the study revealed that the students, overall, reacted positively to the experience of online TBL. The students pinpointed some technical aspects or functions that caught their attention the most, including: setting confidence levels on the MCQs, seeing each other’s answers as a trigger to discussion, and the value of the collaborative approach to the case discussion. They valued the presence and contribution of facilitators, and perceived that the softwares used helped to enhance their overall experience by creating common ground, enhancing teamwork and collaboration, maximising engagement, observing similarities and differences, and getting triggered and feeling encouraged to discuss matters. Accordingly, five themes of text fragments, as illustrated in the Figure 4, emerged:

### Equivalency of Experience

In relation to the nature of the online experience, as an alternative to the face-to-face, classroom experience, the students did not seem to feel deprived of the classroom experience. Some students commented on the benefits of not having to commute to the university. In this context, aware of the emergency situation, they saw the benefits in the immediate situation, its transferability across disciplines, and its sustainability for future use, even when the pandemic settles down.

**01:** “...it was the best possible alternative to face-to-face interactions. It provided me with all the tools that I needed to perform my TBLs...I think at the current stage it can fulfil all our needs...’

**17:** “...I suggest the use of LAMS over in person sessions...”

**18:** “...I would recommend using this software in the future whether in family medicine or other year 4 rotations...”

### Efficiency and Ease of use

Several students commented on the efficiency of the transition, and of the online platform relative to the face-to-face experience. The transition was stressful for all concerned parties, many in lockdown in their homes and students were living not just in Dubai, but throughout the Emirates, and in the last rotation one student was stranded in Sudan. Efficiency thus spelled a reduction in unnecessary stress.

**04:** “...it had all the amazing options which enabled us to run the TBL experiences smoothly...”

**07:** “...seamless moving between the process....”

**13:** “...it is easy to use and fast...’

**11:** “...the format is user friendly and efficient...”

**18:** “...it was a very good software overall (in terms of participation), and made the TBL the most efficient and easy way to work with this method through online learning”

### Organisation

In order for TBL to work online, it must be just as organised as in the classroom setting, otherwise there will be frustrations due to delays and potential for disengagement. Several students commented specifically on organisation and ease of use, either in relation to individual aspects (being able to see own and others’ answers), or in terms of the TBL process and timing which helped to keep everyone together despite the distance.

**12:** “...(I valued) the organisation around the iRATand tRAT, and (application) exercise...”

**16:** “...it is all so organised, and allowed me to view my answers, and to compare them within the group, and across all the groups...’

**15:** “...it was organised, and made sure we are all on the same track...”

### Novelty

In classroom TBL, the students had not previously been able to select how confident they were about their answer in iRAT. They noted they liked this feature which was new to them. In the tRAT, they were able to see the confidence levels and discuss them among each other, triggering discussion about why there were differences within any one group. Students chose to use the confidence meter in differing ways, for example for a rough idea of confidence (0, 50, or 100% increments), or for more fine distinctions, with 10 or 20% increments.

**4:** “...it was a great new experience...”

**5:** “...(I really valued) the fact that we could record confidence levels...”

**6:** “...(it was very beneficial) to get to select our confidence percentage...”

**11:** “...the confidence meter was unique and interesting; it solicited a lot of discussions...”

Moreover, the ‘burning questions’ feature allowed teams to record questions for the facilitator and other students about answers they were uncertain about which triggered more discussion after TRAT. Rather than feeling daunted by seeing their own or each other’s answers, the students commented on the benefits they perceived in benchmarking themselves against others. One student in the fourth rotation felt worried about the fact that others could see their identified answers and briefly dropped out. After some counselling from the discipline lead to understand the purpose of this feature, and getting some feedback from the group, it was agreed that the feature would be kept as it triggered discussion. This was supported by the end of rotation feedback where no one felt concerned about the feature.

**8: “...**we could see each other iRAT answers and confidence levels, and this facilitated discussions; we could ask burning questions...”

**14: “...**seeing everyone’s answer was among the highlights of the overall experience...”

**17: “...**allows me to see everyone’s answers to trigger discussion...”

### Design

The students indicated that the platform design fulfilled its intended function to promote teamwork and collaboration, and was flexible enough to be changed in response to student feedback during the sessions.

**5:** “...the constant effort of Mr. EG’s efforts in improving the case discussions section, where we all had the chance to type in our input added a lot of value to the experience...”

**7:** “...it is a very-well designed platform, seamless moving between the process...I liked the ability to have multiple editors at the same time...’

The students also noted some minor difficulties, that they faced at the beginning of their experience, indicating the existence of a learning curve.

**18: “...**the movement between steps was a bit confusing initially, but I eventually got the hang of it...it was sometimes challenging to input the text but I liked that we all were able to do that...”

They also identified a few opportunities for improvement.

**13: “...**it would have been useful to provide the tutorial beforehand...”

A lot of this constructive feedback focused on improving the application case document, where they ask for this document to be made ready for use, and for them to have control over the respective document and its content.

**3: “...**allow more multimedia to be inserted...”

**6:** “...set a time limit which could be viewed by the students on their screen directly, instead of having the instructor remind us of the time remaining...”

**7:** “...adding pictures, screenshots to the answers of the cases...”

**8:** “...give more formatting options ...adding images to the discussion section...colouring and highlighting the text of the discussion section...”

**10:** “...to be able to add images and graphs in the case discussion box...the ability to add another team leader if need be...video access for a real-life experience...”

## Discussion

This study demonstrated that combining videoconferencing and lesson delivery software together for TBL enables remote facilitation and completion of the Family Medicine curriculum, and that the students value this experience. Although our students and facilitators were spread across geographical locations, they were all able to work collaboratively in real time. The students completed their curriculum requirements, and remained occupied, and connected with their colleagues, Discipline Lead, and other facilitators throughout the rotation. It was also possible, despite the distance, to provide a supportive environment for the students, as well as flexibility for Adjunct Faculty whose timetables had been up-ended by redeployment to front-line COVID-19 duties.

In recent published work, comparing online versus face-to-face TBL, students performed just as well in assessments, but expressed a preference for face-to-face TBL. This negative perception was attributed to some lack of familiarity with the platform which was used, and a lack of warning of an online component ( DeMasi, Harvan and Luca, 2019). The supportive element combined with the special circumstances of the pandemic, in the case of this study, may have contributed to the very positive perception of the experience by the students because it enabled connectivity and continuity in an otherwise anxiety-provoking situation.

### TBL Design and the Community of Inquiry framework

The COI framework provides a useful medium for discussing the online implementation of TBL and how it can be improved in future iterations. The COI framework consists of three, interconnected factors: social presence, teaching presence and cognitive presence ( Vaughan and Garrison, 2005).

### Social presence

The implementation of the online TBL design was deliberate in ensuring as seamless a transition as possible for our students. Multiple communications from LJ prior each rotation beginning helped to set expectations and provided clear instructions to students on how and where to access materials and sessions. Introductory session on LAMS (EG), pilot of MS Teams and initial ice breaker (LJ) helped to develop knowledge and build trust community between the students and faculty. Ice breaker sessions especially helped to inform the design of groups to help enable all student voices to be heard.

As illustrated in the framework that was generated as part of this study, the students emphasized the value and equivalency of the experience, and how they were able to remain connected throughout the journey. The students were satisfied with the efficiency and ease-of-use of the platform, and the overall organization of the online TBL, and felt their voice was heard within and in between sessions. The students also felt they had a voice to suggest further improvements, of which many were suggested as noted in the design theme of this framework.

### Teaching presence

COI advocates for a teaching presence that is not central to the faculty, but as a role in which both students and faculty can assume at different points in the process. This enables student-centricity enabling students to assume more control of their learning. In our online implementation of TBL, student groups chose a team leader who assumed the teacher role and helped to cohere the group feedback and facilitate small group discussion. Throughout the sessions, core faculty acted as facilitators of discussions, using the feedback gathered from the LAMS software to guide discussions amongst the entire group of students.

The design theme of the framework where the interface seemed to resonate well with the students, which can be related to both the social and teaching presence factors of the COI. During the beginning sessions, EG introduced and facilitated using the LAMS components which helped students feel supported in using the system and in getting comfortable with the online environment. Channels in MS Teams were utilized for small group discussions, which enabled LJ and EG to move between groups and facilitate any problems or stimulate discussion. Gradually throughout the sessions, EG and LJ were able to step back a bit as students became more autonomous and began to drive their own group discussion and develop their own methods of documenting their small group discussions. The role of the Discipline Lead (LJ) in facilitating the TBL process also to support team spirit with the students during the particular difficulties created by remote working during the pandemic.

### Cognitive presence

The cognitive presence, which is the extent to which learners are able to construct meaning and think critically through their participation in the community, was facilitated by both the features of the platforms used and the design of the TBL sessions themselves. The iRAT and tRAT stages of TBL serve as triggering events to inquiry and through breaking students into small groups in which one member assumes a leadership role, enables opportunities for active discourse. Finally, regrouping the entire class for further discussion promotes continues the exploration and integration of knowledge.

Student responses relating to cognitive presence were largely expressed through the differing unique attributes of the platform such as: confidence rating and burning questions, which introduced novelty to the experience. The setting of confidence levels on the MCQs was particularly highlighted among both students and teachers as a unique measure to demonstrating levels of understanding. This feature was unique to the TBL online sessions and facilitated by the LAMS software. The collaboration approach to the case discussions was also noted by students as valuable to their TBL experience.

### Learning Points for Future Implementation

Based on students’ perception, the combination of the videoconferencing and lesson delivery software (LAMS) served to improve the TBL experience. In reviewing published advice from the Team Based Learning Collaborative ( Clark *et al*, 2018) which discusses several best practice tips for online TBL, we found parallels to our experience. These included: the importance of not skipping the step of good orientation to the TBL process at the start, maintaining the integrity of the iRAT, and fostering good team collaboration through interactions in smaller groups. The group formation step was preserved particularly for the final group which never met in person and was achieved just as in the classroom including with the creation of group accountability rules. Peer-evaluation has been noted to transfer readily to online platforms, and this was evident in the case of this study. iRAT/tRAT questions could be locked in the e-learning platform once students had completed the discussions in order to ensure the preservation and security of the questions for future use. Discipline-specific facilitators were able to bring Family Medicine to life, using the application exercise. The e-learning software enabled the application exercise results not only to be reported together, but also for the online documents to be preserved for revision purposes. While the transition between whole class and team levels is rather trivial in an on-site classroom, in the online scenario there were many possible obstacles.

### Technology and training

Significant time and effort went in to ensuring that faculty and students were able to connect and undertake online TBL. The Discipline Lead had to ensure all Adjunct Faculty, who had to join as ‘guests’ into the system, had proper initiation into the system and could connect successfully. This often entailed a trial run before the actual TBL session. During sessions, internet connections could sometimes vary in stability and often was corrected by students turned their cameras off, which may have either helped or hindered engagement.

One advantage of using the videoconferencing platform was that it was tightly integrated with other applications in use by the University, including the calendar function, which made timetabling of meetings simple and transparent. This was especially helpful with our rotation 5 students, many of whom were fasting during the holy month of Ramadan and the need to accommodate the timetabling was necessary.

Students also responded positively to the video conferencing system noting that they saved time in commuting to campus and saved paper by not printing.

### Organization

Many features of the videoconferencing software overlapped with that of the University’s Learning Management System (LMS), including file storage and document retrieval. During the online TBL sessions, many documents and resources were shared with students via the video conferencing system. However, the LMS was the central repository for students to retrieve important documents such as their core course reading materials. To ensure clarity for the students, the Discipline lead monitored and transferred information which supported overall learning outcomes from the videoconferencing system, into the LMS.

### Limitations and Future Direction

This study is characterized by several limitations. To start with, although this study reflected upon an important transition and offered plenty of in-depth insights to what took place, the investigation revolved around a single institution, in a very particular time. Hence, the generalizability of the findings is limited to situations that are comparable to that of this study. Moreover, the target population was limited to the students who underwent the respective clerkship online (n=19), out of which 18 voluntarily chose to participate. It is recommended for future studies to include a larger sample size, across several higher education institutions. It would also be valuable to complement the students’ narrative with quantitative data that tests the efficacy of the transition, be it with a pre-post assessment or otherwise.

## Conclusion

This study offers a unique demonstration of the value of agility in responding to a crisis, and illustrates the advantages of TBL in promoting analytical and self-directed learning even in extreme circumstances. Rather than ‘dumping’ lecture content on an online platform, with no interaction with students, the TBL sessions allowed the facilitators, including the Discipline Lead, to get to know the students, on a personal level, monitor and evaluate their performance, over time, facilitate real-time discussions, and enable effective student progression.

## Take Home Messages

• TBL content and structure can be seamlessly transferred from the classroom setting to an online environment.
• Transitioning to an online platform that supports a learner centric process can enhance the TBL experience for students.
• The Community of Inquiry provides a useful framework to design and evaluate effective online TBL learning experiences.
• Working in channels on a conferencing platform facilitated teamwork.
• With clear central coordination in distance learning, it is possible for all stakeholders to promptly adapt to change.

## Notes On Contributors

**Dr. Lisa Jackson** is Associate Professor- Family Medicine, College of Medicine, Mohammed Bin Rashid University of Medicine and Health Sciences, Dubai, UAE. ORCID ID: https://orcid.org/0000-0003-4161-6031

**Ms. Farah Otaki** is Senior Specialist- Strategy and Institutional Excellence Department, Mohammed Bin Rashid University of Medicine and Health Sciences, Dubai, UAE. ORCID ID: https://orcid.org/0000-0002-8944-4948

**Ms. Leigh Powell** is Senior Learning Designer, Institute for Excellence in Health Professions Education, Mohammed Bin Rashid University of Medicine and Health Sciences, Dubai, UAE. ORCID ID: https://orcid.org/0000-0001-6714-7371

**Mr. Ernie Ghiglione**, Project Manager, LAMS International, Singapore.

**Professor Nabil Zary** is Institute for Excellence in Health Professions Education, Mohammed Bin Rashid University of Medicine and Health Sciences, Dubai, UAE. ORCID ID: https://orcid.org/0000-0001-8999-6999

## Declarations

Ernie Ghiglione is affiliated with LAMS International and has acted as a researcher in this study protocol.Lisa Jackson, Farah Otaki, and Nabil Zary have no declarations of conflict of interest.
