# Supplementary material for: Study of a COVID-19 induced transition from Face-to-Face to Online Team-Based Learning in Undergraduate Family Medicine
Source: MedEdPublish (2016). 2020 Oct 16;9:232. [Version 1] doi: 10.15694/mep.2020.000232.1 (PMC10697564; doi:10.15694/mep.2020.000232.1)
Supplement: Peer review history

# Reviewer report: Reviewer response for version 1

**DOI:** 10.21956/mep.20159.r31146

**Published:** 2020-10-26

**Authors:** Barbara Jennings

**Affiliations:** 1 Norwich Medical School

This review has been migrated. The reviewer awarded 4 stars out of 5 In a recent commentary (Hays, R. et al. MedEdPublish, 9, [1], 142, https://doi.org/10.15694/mep.2020.000142.2) the editorial team commented on 39 articles describing adaptations that clinical educators had made in response to the first wave of the covid-19 pandemic. One of our conclusions was that we need research to evaluate the new strategies described. In this article by Jackson et al., research findings are presented about a pandemic-related adaptation at a medical school in UAE, and it is a timely response to the community’s need for data to drive curriculum development. The authors have completed a qualitative study, and thoroughly analysed their evaluation of online Team-based learning (TBL). In their introduction, the authors discuss principles of TBL and also appraise published evidence for its use as a valuable part of a medical curriculum and in particular recent applications of online versions of TBL. Readers will no doubt find the bibliography very useful in itself.In their evaluation, the authors reflect on the move to online TBL with respect to a community of enquiry model, exploring social presence, teaching presence, and cognitive presence. Although, this research must have been organised rapidly and conducted at a challenging time, research governance steps were thorough, and efforts to avoid bias are explained in the data collection section. Also, I noted the student-centred educational environment e.g. logistical adjustments based on learners’ needs.The writing is very engaging throughout, and the figures are helpful. I wish more articles had a study timeline illustrated with the clarity of figure 3.I have no expertise in the methodology and conventions for qualitative phenomenological research, so I have a question for the authors; how complete is the data presented in the results/analysis section – are the comments included comprehensive or representative?Another question, relates to conclusions around the use of video-conference platforms that allow break out rooms and multiple channels – do the authors think this could present opportunities for more efficient use of faculty time/ teacher presence in small group settings such as TBL and PBL?In the discussion, the authors outline one or two limitations that I had noted about the study design (e.g. a small cohort) and some of these were inevitable given that the research emerged from a natural experiment etc. I agree with a previous reviewer, that some of the abbreviations and terms could have been elaborated on as they were introduced.Overall, I really enjoyed reading this thought-provoking article and think there are generalizable findings for many other types of synchronous, online small group learning. Several sections resonated with my own experiences of using digital/online PBL since March and I will recommend it widely to facilitators and curriculum leads.

Reviewer Expertise:

NA

No decision status is available

# Reviewer report: Reviewer response for version 1

**DOI:** 10.21956/mep.20159.r31147

**Published:** 2020-10-20

**Authors:** P Ravi Shankar

**Affiliations:** 1 American International Medical University

This review has been migrated. The reviewer awarded 5 stars out of 5 This is an interesting and well-written manuscript on online TBL in a medical school in the United Arab Emirates. The authors have described the TBL process and the process followed during the study in detail. The diagrams help the reader understand the process. My suggestions for further strengthening the manuscript are: There are many abbreviations in the Abstract which can be expanded on first use in the manuscript. The authors should clarify that EG, LJ and LP mentioned refers to the authors of the study. I was confused for some time regarding this. I believe Sudan should be two hours behind Dubai. Time zones are a challenge to be addressed during online learning. What exactly do the authors mean by a discipline lead? How is TBL organized at the university? The modifications to the online TBL carried out at the university can be detailed. The authors can explain the confidence meter in greater detail. Also, the burning questions feature and the fact that each participant could see other’s answers during the iRAT.The student numbers are low, and the increased attention focussed by faculty and support staff on the students may have been partly responsible for their positive perception.

Reviewer Expertise:

NA

No decision status is available
